# Development of a Nursing Practice Framework for 5G‐Enabled Cross‐Regional Remote Surgery

**DOI:** 10.1155/jonm/9403452

**Published:** 2026-10-04

**Authors:** Yaling Wang, Shuang Jin, Lili Chen, Yang Yu, Xiaofang Chen, Lan Jiang, Xiaodan Wu, Gang Chen, Hua Cao

**Affiliations:** ^1^ Department of Operating Room Fuzhou University Affiliated Provincial Hospital Fuzhou China

**Keywords:** cross-regional nursing, distributed healthcare, nursing theory, remote surgery, robotic surgery

## Abstract

**Objective:**

Established perioperative and nursing theories provide foundations for patient safety, adaptation, caring, communication, and professional practice. This study aimed to develop a context‐specific operational framework that applies and specifies these concepts for 5G‐enabled cross‐regional remote surgery, particularly in relation to technology‐mediated care, distributed collaboration, and adaptive nursing capacity.

**Methods:**

This mixed‐methods study combined a systematic literature review (four databases; topic search, January 2000‐July 2025; foundational theory searches unrestricted) with case analysis of two cross‐continental, three‐site 5G‐enabled robotic surgeries. The review examined how established nursing theories and perioperative practice guidance have been applied to remote, robotic, and technology‐mediated surgical contexts. Literature synthesis and case analysis were integrated to identify operational domains for framework development.

**Results:**

Established nursing theories remained useful conceptual lenses for understanding patient safety, healthcare‐system interaction, adaptation, communication, professional learning, and human responses to technologically advanced care environments. Case analysis did not indicate theoretical obsolescence; rather, it identified areas requiring context‐specific operational specification in remote robotic surgical practice. The AORN perioperative patient‐focused model, Roy’s adaptation model, Benner’s skill acquisition model, and related theories informed the proposed framework. In the two cases, remote nursing involved technological complexity, independent patient‐site teams, and substantial interprofessional coordination. The proposed framework incorporates three interrelated components: (1) technology‐mediated nursing, emphasizing integration of advanced technological systems into nursing judgment and care processes; (2) distributed nursing collaboration, addressing teamwork across geographically separated sites; and (3) nursing adaptive capacity, underscoring continuous learning and flexible response in dynamic technological environments. The preliminary framework may provide guidance for preoperative planning, intraoperative execution, and postoperative care in remote robotic surgery.

**Implications for Nursing Management:**

The preliminary framework may inform perioperative nurse managers, nursing directors, and operating room leaders in patient‐site staffing and role allocation, training and competency assessment, standardized communication protocols, emergency drills, quality indicators, and distributed workflow oversight.

**Conclusion:**

The framework offers a preliminary, context‐specific operational foundation for cross‐regional remote robotic surgery nursing. It applies, specifies, and supplements established nursing theories rather than replacing them and may support process standardization and care‐quality evaluation. Further multicenter validation, instrument development, and outcome‐based evaluation are needed to refine its applicability.

## 1. Introduction

Surgical care inequality remains a major global health challenge. The Lancet Commission on Global Surgery estimates that approximately 5 billion people worldwide lack timely access to safe and affordable surgical care [1]. Geographic barriers, specialist shortages, and resource limitations in remote and underserved regions contribute substantially to preventable morbidity and mortality [2]. Conventional surgical care models, dependent on co‐located multidisciplinary teams and advanced equipment, have proven inadequate in addressing these disparities, particularly in low‐ and middle‐income countries [3]. Cross‐regional remote surgery has emerged as a promising strategy, leveraging advanced robotic systems and high‐speed telecommunications to enable expert surgical teams to perform procedures across geographic boundaries [4]. Recent successful implementations, including transcontinental operations spanning over 6000 km with acceptable latency, have confirmed both technical feasibility and clinical outcomes comparable to conventional surgery [4].

However, the rapid evolution of remote surgical technology has outpaced the development of context‐specific nursing practice guidance [4]. Cross‐regional remote surgery involves geographically distributed teams connected via telecommunication and robotic technologies, placing new demands on nurses to coordinate patient care across multiple sites, technological platforms, healthcare systems, and cultural contexts [5]. While pioneering cases have established technical feasibility, the nursing implications of distributed surgical care remain incompletely specified. Current literature focuses largely on technical specifications and surgical outcomes, with limited attention to coordination and patient‐care challenges faced by nursing teams [6]. Existing perioperative and nursing theories, including the perioperative patient‐focused model, remain highly relevant to patient safety, physiological and behavioral responses, nursing care, adaptation, caring, communication, and healthcare‐system interactions. The central question is therefore not whether these theories should have anticipated contemporary technologies, but how their enduring concepts can be applied and operationalized across geographically distributed teams, networked robotic systems, and cross‐institutional workflows [7]. This need is evident in challenges such as sustaining therapeutic relationships through technological interfaces, aligning care processes across different regulatory systems, and navigating cross‐cultural team dynamics [8].

This study addresses this practice guidance gap by developing a cross‐regional surgical nursing framework (CRSNF) informed by systematic literature review and analysis of two transcontinental, trisite remote robotic surgery cases. The objectives are threefold: (1) to identify core operational domains essential for nursing practice in distributed surgical environments through literature synthesis and clinical case analysis; (2) to examine relationships and interactions among these domains in light of established theoretical concepts and empirical evidence; and (3) to propose a preliminary, practice‐oriented framework to guide evidence‐based nursing practice, curriculum development, and quality evaluation protocols in cross‐regional remote surgery. The CRSNF aims to provide operational guidance grounded in established nursing theory while highlighting priorities for future empirical research and clinical innovation.

## 2. Methods

### 2.1. Study Design

This study utilized a mixed‐methods design, combining systematic literature review and case analysis to develop the CRSNF. The research followed a three‐phase approach for framework development: (1) systematic literature review to identify the existing knowledge base and practice guidance gaps, (2) case analysis to extract practice insights from remote robotic surgical nursing, and (3) theoretical synthesis to construct an operational framework grounded in established nursing concepts.

### 2.2. Systematic Literature Review

#### 2.2.1. Search Strategy

A comprehensive systematic literature review approach was adopted, searching PubMed, CINAHL, Cochrane Library, and Web of Science databases (Figure 1). The topic‐specific search for remote and robotic surgical nursing covered January 2000 to July 2025. Targeted searches for foundational nursing theories used in framework development were not restricted by publication year. Keyword combinations included the following: “remote surgery,” “telesurgery,” “robotic surgery,” “nursing care,” “surgical nursing,” “distributed healthcare,” and related theory terms, combined using Boolean operators.

**FIGURE 1:**
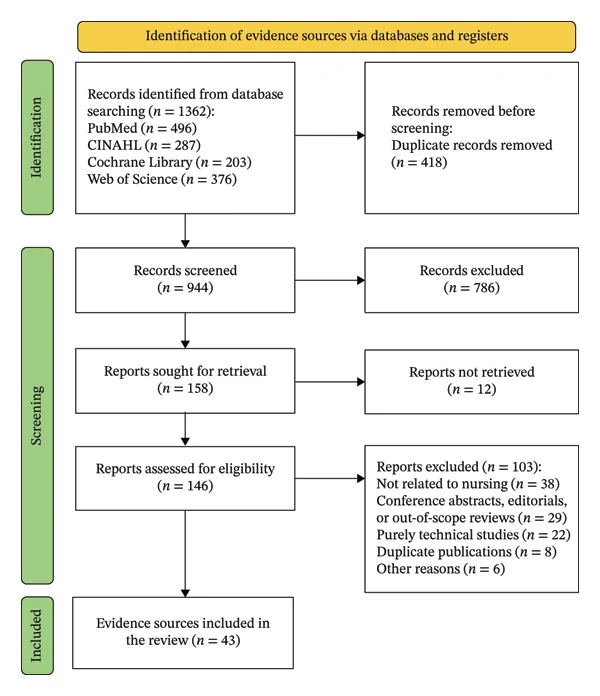
PRISMA 2020 flow diagram for evidence‐source identification and selection in the systematic literature review. Records were identified from PubMed, CINAHL, Cochrane Library, and Web of Science. The topic‐specific search covered January 2000–July 2025, whereas targeted searches for foundational nursing theories were not date restricted; together, the searches yielded 1362 citations. After removal of 418 duplicates, 944 records underwent title and abstract screening; 786 were excluded. Full texts were sought for 158 reports, of which 12 could not be retrieved. Of the 146 reports assessed for eligibility, 103 were excluded: not related to nursing (*n* = 38), conference abstracts, editorials, or out‐of‐scope reviews (*n* = 29); purely technical studies (*n* = 22); duplicate publications (*n* = 8); and other reasons (*n* = 6). Forty‐three evidence sources met the inclusion criteria and were retained for the review.

#### 2.2.2. Inclusion and Exclusion Criteria

Inclusion criteria: (1) peer‐reviewed empirical studies, evidence syntheses, theoretical or conceptual papers, practice guidance, or expert consensus; (2) sources relevant to remote or robotic surgical nursing or to theoretical domains used in framework development; (3) literature published in English or Chinese; and (4) sources providing extractable empirical, theoretical, or practice‐relevant content.

Exclusion criteria: (1) conference abstracts, editorials, and out‐of‐scope reviews; (2) purely technical sources without nursing or framework relevance; and (3) duplicate publications.

#### 2.2.3. Literature Screening and Narrative Methodological Assessment

Two independent researchers screened the literature according to predefined criteria. Screening disagreements were resolved through discussion or, when necessary, consultation with a third reviewer. Following source selection, the review team conducted a narrative methodological assessment. No formal standardized quality‐appraisal or risk‐of‐bias instrument and no numerical quality score were applied to the 43 included evidence sources. The narrative assessment considered source type and design, clarity of purpose, transparency of methods or argumentation, completeness of reporting, and relevance to remote or robotic surgical nursing and framework development. No source was excluded solely on the basis of this assessment.

### 2.3. Case Analysis

Analysis focused on nursing practice experiences from two transcontinental, trisite remote robotic surgeries conducted at our institution. These procedures provided first‐hand data for exploring the complexities and challenges of cross‐regional remote surgical nursing. To descriptively characterize case‐level nursing indicators, three self‐developed assessment tools were employed: (1) patient safety indicators [9] and established nursing quality metrics [10], (2) measures of nursing team adaptability, and (3) indicators of medical–nursing collaboration [11]. Given the two‐case sample and the use of self‐developed measures, all case findings were treated as exploratory and descriptive; no inferential statistical testing or effectiveness comparison was performed.

### 2.4. Framework Development and Theoretical Synthesis

Literature and case findings were synthesized to identify core operational domains and relationships in cross‐regional remote surgical nursing. The resulting preliminary model was designed to translate established nursing concepts into practical guidance for robotic surgery nursing practice. Methodological considerations informed the narrative synthesis: findings supported across multiple sources were prioritized, whereas isolated findings from less clearly reported or less directly relevant studies were interpreted cautiously and used mainly to identify contextual issues or research gaps.

## 3. Results

Narrative methodological assessment summary: the source evidence base showed substantial heterogeneity in source type, study design, clinical context, and reporting. Because no formal appraisal tool was used, no source‐level risk‐of‐bias ratings or common numerical quality summary were available. Most sources stated their aims and practice contexts clearly, but methodological and reporting detail varied. Sources with clearer methods or arguments and more direct relevance to remote or robotic surgical nursing were given greater interpretive weight. Isolated, less clearly reported, or indirect sources were used primarily to identify contextual issues, theoretical considerations, or research gaps. The absence of a formal design‐specific appraisal tool and source‐level appraisal table is a methodological limitation of the review.

### 3.1. Application and Contextual Specification of Established Nursing Theories

Perioperative and nursing theories, including the AORN perioperative patient‐focused model, Orem’s self‐care theory, Roy’s adaptation model, Watson’s theory of human caring, Benner’s skill acquisition theory, Henderson’s need theory, Donabedian’s quality assessment model, Leininger’s transcultural care theory, and the technology acceptance model (Table 1), continue to provide foundational lenses for understanding patient care, nursing practice, adaptation, communication, professional development, and human responses in technologically advanced surgical environments. The analytic question in this study is therefore not to judge these theories by whether they contain technology‐specific constructs, but to examine how their core concepts can be applied, specified, or supplemented in cross‐regional remote robotic surgery.

**TABLE 1:** Application and contextual extension of established nursing theories in cross‐regional remote robotic surgery.

Theory category	Specific theory	Core concepts	Contextual application, extension, or supplementation in remote robotic surgery
Patient‐centered nursing theory	AORN perioperative patient‐focused model [12]	Patient‐centered perioperative care; patient safety; physiological and behavioral responses; healthcare‐system interaction	Apply the existing patient‐safety and healthcare‐system domains to distributed workflows by specifying network reliability, data integrity, equipment readiness, cybersecurity, remote operation precision, and multisite communication responsibilities
	Orem’s self‐care theory [13]	Self‐care need identification and nursing support	Apply self‐care assessment and education to technology literacy, tolerance for network interruptions, and understanding of remote medical information

Adaptive nursing theory	Roy’s adaptation model [14]	Promoting individual adaptation through stimulus identification	Apply adaptation concepts to both patients and nursing teams, including adaptation to technical operations, system monitoring, remote coordination, and changing team responsibilities
	Neuman systems model	Promoting adaptation through system stability	Apply system‐stability concepts to technological stressors, including network interruption, equipment malfunction, cybersecurity risk, and failure‐response mechanisms.

Skill development theory	Benner’s skill acquisition theory [15]	Linear development model from novice to expert	Operationalize professional development in a rapidly changing technological environment by combining perioperative expertise with robotic‐system familiarity, troubleshooting, and cross‐site communication skills.
	Henderson’s need theory [16]	Meeting 14 basic human needs	Apply human‐needs assessment to technology‐related information needs, understanding of remote team roles, and reassurance regarding technology‐mediated care

Relationship nursing theory	Peplau’s interpersonal relations theory [17]	Establishing therapeutic relationships	Apply interpersonal relationship concepts to virtual and technology‐mediated communication, including trust building, emotional support, and therapeutic presence across distance
	Watson’s caring theory [18]	Humanistic caring practice	Apply caring practice to technology‐related anxiety, data security, trust in remote systems, and virtual presence as elements of patient‐centered caring

Cross‐cultural nursing theory	Leininger’s transcultural nursing theory [19]	Cultural sensitivity and cultural appropriateness in nursing	Apply cultural‐sensitivity principles to multicultural, cross‐regional teams while considering privacy expectations, technology acceptance, digital literacy, and family decision‐making traditions
	King’s goal attainment theory [20]	Goal setting and collaborative achievement	Operationalize goal attainment through multiparty remote goal setting, shared situational awareness, and mechanisms to maintain goal consistency across sites.

Quality management theory	Donabedian’s quality evaluation model [21]	Three‐dimensional evaluation: structure–process–outcome	Specify structure, process, and outcome indicators for remote surgery, including network infrastructure, robotic platforms, communication protocols, data security, and cross‐regional quality monitoring
	Technology acceptance model [22]	Predicting perceived usefulness and ease of use	Supplement individual acceptance factors with sociotechnical variables, including team collaboration, organizational support, cross‐regional coordination, cultural differences, and distributed responsibility

#### 3.1.1. AORN Model Application and Contextual Specification

The AORN perioperative patient‐focused model is not limited to co‐located perioperative practice. It provides a relevant foundation for remote robotic surgery because its domains encompass patient safety, physiological and behavioral responses, nursing care, and healthcare‐system factors. In remote surgery, the model can be operationalized by specifying how patient‐safety and healthcare‐system domains address network reliability, data integrity, cybersecurity, equipment readiness, remote operation precision, and communication across geographically separated surgical sites.

#### 3.1.2. Contextual Specification of Self‐Care, Human Needs, and Quality Evaluation Theories

Orem’s self‐care theory can inform assessment of patient and family technology literacy, tolerance for network interruptions, and understanding of complex remote medical information [23]. Henderson’s 14 basic needs can similarly guide assessment of technology‐related information needs and understanding of remote team roles. Donabedian’s structure–process–outcome model remains useful for quality assessment, while its structure and process domains can be specified to include network infrastructure, robotic platforms, data security, cross‐regional coordination mechanisms, remote operation protocols, and real‐time communication procedures [24].

#### 3.1.3. Contextual Specification of Relational, Adaptation, and Cultural Theories

Watson’s caring theory can be applied to technology‐mediated contexts by incorporating technology‐related anxiety, data security, trust in remote systems, and virtual presence as components of caring practice. Roy’s adaptation model can be applied not only to patients but also to nursing teams who must adapt to technical operations, system monitoring, and remote coordination. Leininger’s transcultural care theory remains important for coordinating multicultural teams while respecting diverse values, including religious beliefs, family decision‐making traditions, privacy expectations, technology acceptance, and digital literacy differences [25].

#### 3.1.4. Skills and Technology Acceptance

Benner’s novice‐to‐expert framework remains valuable for understanding professional development, while remote robotic surgery requires this progression to be operationalized in a rapidly changing technological environment [26]. Nurses require simultaneous development of perioperative judgment, robotic‐system familiarity, failure‐response capability, and cross‐site communication skills. Technology acceptance models also provide useful insight into perceived usefulness and ease of use [27], but in remote surgery, they can be supplemented with sociotechnical factors such as team collaboration, organizational support, cross‐regional coordination, cultural differences, and distributed responsibility.

#### 3.1.5. Additional Conceptual Applications

Peplau’s theory can inform therapeutic relationships in virtual and technology‐mediated environments [28], King’s theory can guide multiparty remote goal setting and collaborative achievement [20], and Neuman’s model can support assessment of technological stressors and protective mechanisms [29]. Accordingly, remote robotic surgery calls for three forms of contextual operationalization: integrating technological dimensions into nursing assessment and care processes, specifying human–machine collaboration within nursing work, and systematizing interdisciplinary competencies for distributed surgical care.

### 3.2. Case Analysis

#### 3.2.1. Basic Case Information

This study analyzed two cases of cross‐regional, trisite 5G‐enabled remote 3D naked‐eye robotic surgery conducted at our institution using the KangDuo endoscopic surgical robot system (SR2000). Case 1 involved a radical prostatectomy, and Case 2 involved a urethral reconstruction. Both procedures adopted a multinode collaborative architecture linking France, Beijing, and Fuzhou, with patients located in Fuzhou and surgical tasks jointly performed by surgical teams across the three sites. Both procedures were completed, with surgical durations of 268 min and 180 min, respectively. The recorded network parameters are presented in Table 2, and no technical interruption affecting surgical progress was documented in either case.

**TABLE 2:** Recorded technical parameters in the two cases.

Technical indicator	Value (ms)	Notes
Beijing to Fuzhou network latency	Average 48	0% packet loss
France to Fuzhou network latency	Average 216	0% packet loss
Fuzhou to Beijing video transmission latency	109	—
Fuzhou to France video transmission latency	193	—

#### 3.2.2. Nursing Team Configuration and Role Analysis

Both patient site and remote sites were equipped with professional technical personnel responsible for equipment monitoring and technical support, but these were not part of the nursing team scope (Table 3).

**TABLE 3:** Nursing team personnel configuration.

Location	Nursing role	Number	Primary responsibilities
Patient site	Circulating nurse	1	Overall coordination, communication with remote physicians, and environment management
Patient site	Scrub nurse	1‐2	Instrument passing and robotic arm replacement and installation

#### 3.2.3. Characteristics of Nursing Practice Environment

##### 3.2.3.1. Technological Environment Complexity

The operating room deployed multiple technical equipment systems including robotic surgical systems, high‐definition audio–video communication systems, and patient monitoring systems, forming a complex technological ecosystem environment. Nursing personnel needed to adapt to this technology‐intensive work environment while maintaining traditional nursing professionalism and learning to work collaboratively with technological systems.

##### 3.2.3.2. Nursing Team Independence Characteristics

Due to the absence of nursing personnel at the remote sites, the patient‐site nursing team needed to independently complete all nursing decisions and nursing operations without direct guidance from remote nursing experts. This independence required the nursing team to possess stronger professional judgment capabilities, emergency response abilities, and collaborative communication skills with remote physicians. The nursing team became the sole nursing professional force at the patient site, bearing full responsibility for ensuring patient safety and nursing quality.

##### 3.2.3.3. Nursing Practice Specificity

Remote communication and coordination became an important responsibility of the circulating nurse, requiring accurate transmission of surgical site information and understanding of remote physician instruction requirements. Scrub nurses needed to accurately understand and coordinate with remote physician operation requirements without face‐to‐face guidance. Nursing decisions needed to be completed independently without support from remote nursing experts, placing higher demands on the nursing team’s professional judgment capabilities.

#### 3.2.4. Descriptive Nursing Practice Findings

Across the two cases, no adverse event was recorded for the selected patient‐safety indicators, and protocol adherence was documented as 100%. From Case 1 to Case 2, technical anomaly handling time was 1 min shorter, the recorded communication response‐time range shifted from 15–20 s to 10–15 s, and the proficiency score was 0.8 points higher (Table 4). These values represent descriptive between‐case differences only and were not subjected to inferential statistical testing. Descriptive collaboration scores ranged from 7.2 to 8.5 out of 10; internal nursing‐team collaboration received the highest score, while robotic‐arm operation skills and technical terminology standardization were identified as areas for further development (Table 5).

**TABLE 4:** Descriptive nursing adaptability indicators in two cases.

Descriptive indicator	Case 1	Case 2	Observed difference
Technical anomaly handling time (minutes)	3.0	2.0	1.0‐min decrease
Remote communication response time (seconds)	15–20	10–15	5‐s downward shift
Nursing operation proficiency score (1–10 points)	7.5	8.3	0.8‐point increase

**TABLE 5:** Descriptive medical–nursing collaboration scores.

Collaboration dimension	Average score (1–10 points)	Observed strengths	Areas for improvement
Nursing team internal collaboration	8.5	Coordination and role clarity	Robotic arm operation skills need strengthening
Collaboration with remote physicians	7.8	Communication timeliness and coordination	Surgical rhythm coordination needs improvement
Technical support team collaboration	7.2	Response speed	Technical terminology needs standardization

#### 3.2.5. Case Summary

In these two cases, nursing practice involved technological complexity, patient‐site team autonomy, and substantial interprofessional coordination demands. Both procedures were completed, and no adverse event was recorded for the selected indicators; however, these observations cannot establish the safety or effectiveness of the framework. The case analysis also identified a need for more explicit, context‐specific protocols that translate established nursing concepts into distributed surgical care.

In the two cases analyzed, nursing teams appeared to rely predominantly on experiential knowledge and real‐time adaptability rather than standardized, theory‐informed operational protocols. These observations informed the proposal of structured guidance that integrates existing nursing theories with the sociotechnical requirements of remote surgical environments.

Taken together, the literature synthesis and descriptive observations from these two cases supported the proposal of a preliminary, context‐specific practice framework integrating foundational nursing theories with the workflow, communication, safety, and adaptation requirements of cross‐regional remote surgery. They do not demonstrate clinical effectiveness or generalizability; rather, they identify priorities for further protocol development and empirical evaluation.

### 3.3. CRSNF

#### 3.3.1. Framework Development Basis and Theoretical Anchoring

Drawing on findings from the systematic literature review and case analysis, established nursing theories were used as foundational conceptual resources for interpreting remote robotic surgical nursing. The CRSNF was developed as a context‐specific operational framework that integrates and specifies these theories for distributed, technology‐mediated surgical environments, comprising three core components: technology‐mediated nursing, distributed nursing collaboration, and nursing adaptive capacity [30].

#### 3.3.2. CRSNF Core Concepts and Definitions

##### 3.3.2.1. Framework Core Conceptual System

Technology‐mediated nursing is a nursing practice enacted through technological systems in remote robotic surgery [7]. Core elements include technology dependence, nursing professionalism, and human–machine collaboration. Nurses function as collaborative partners forming an organic whole with technological systems, exercising professional judgment through technological interfaces while maintaining independent evidence‐based decision‐making and risk management [31].

Distributed nursing collaboration denotes interprofessional coordination via digital platforms [32]. Core elements encompass spatial separation, role redefinition, and coordination mechanisms. Geographic separation requires collaboration mediated entirely through technological platforms. Nursing teams assume traditional responsibilities plus communication with remote surgeons, often as the sole on‐site nursing force [33]. Effective coordination requires standardized communication protocols, information‐sharing mechanisms, and emergency response procedures [34].

Nursing adaptive capacity refers to continuous learning and dynamic adjustment in technologically complex environments [35]. Core elements include technological learning ability, environmental adaptability, and innovative decision‐making. Nurses must rapidly master new equipment and procedures, support consistent quality across diverse technological configurations, and creatively address unexpected problems through risk identification, assessment, and prevention strategies [36].

##### 3.3.2.2. Relationships Between Concepts

The three concepts are proposed as an interdependent system. Technology‐mediated nursing provides a technological foundation, distributed collaboration provides an organizational structure, and nursing adaptive capacity may support the capability dimension. The proposed relationships are intended to facilitate coordination among the three concepts and may contribute to stable system operation.

#### 3.3.3. CRSNF Operational Architecture

##### 3.3.3.1. Framework Overall Structure

The CRSNF is proposed as an integrated three‐dimensional architecture comprising technological, collaborative, and adaptive dimensions and is intended to provide a preliminary practice‐oriented structure for remote robotic surgical nursing [37].

The technological dimension emphasizes nursing professional judgment exercised through technological interfaces, requiring nurses to master technical competencies such as network performance monitoring, equipment status assessment, and data transmission quality control [37]. Human–machine collaborative decision‐making permeates nursing practice, with nurses conducting assessments, identifying risks, and making intervention decisions in partnership with technological systems [38]. This distributed configuration requires nursing teams to translate professional judgment across technological interfaces while coordinating with remote surgeons, often as the sole on‐site nursing force [33].

The collaborative dimension addresses nursing team responsibilities under conditions of spatial separation. Nursing teams may assume greater independent responsibilities while establishing interprofessional collaborative mechanisms with remote healthcare teams [33]. Role redefinition may require nursing personnel to fulfill both traditional nursing functions and communication‐coordination functions with remote lead surgeons. Proposed coordination mechanisms include standardized communication protocols, information‐sharing platforms, and emergency response procedures intended to support effective collaboration among cross‐regional teams. At patient sites, nursing teams may serve as the principal on‐site nursing representatives, with responsibilities for communicating nursing needs and supporting nursing quality monitoring [31].

The adaptive dimension focuses on continuous capability development. Technological learning ability requires nursing personnel to continuously master new equipment operations, new procedural standards, and new technological applications, establishing dynamic updating mechanisms for personal technological capabilities. Environmental adaptability is reflected in nursing teams′ capacity to support consistent nursing quality under different technological configurations, network conditions, and collaborative modes [35].

##### 3.3.3.2. Framework Applicability Boundaries

The framework’s applicability scope is primarily limited to cross‐regional remote robotic surgical nursing practice fields, including geographic spatial separation, technological system mediation, and independent nursing team operations.

### 3.4. Implications for Nursing Management

#### 3.4.1. Nursing Process Standardization Design

Technical infrastructure standards specify 5G network latency as ≤ 100 ms (optimal) or ≤ 300 ms (acceptable); bandwidth 200 Mbps–1.4 Gbps depending on video resolution and system complexity; network reliability “seven nines” (99.99999%) with packet loss of < 0.012%; and backup connectivity via satellite and redundant fiber optic links [39].

Distributed nursing collaboration standards build upon Chinese Expert Consensus on Robotic Surgery Nursing Coordination, extending to cross‐regional practice [40]. Standards emphasize clear role definitions, delineating communication boundaries with remote surgeons, and standardized protocols for information‐sharing and emergency response via cloud‐based platforms [41].

Nursing adaptive capacity development standards establish technological learning assessment systems (new equipment certification and continuing education), environmental adaptability evaluation across technological configurations, and innovative decision‐making processes incorporating risk identification and prevention measures.

Quality management standards establish real‐time feedback loops collecting vital signs, equipment status, and team communications; standardized risk assessment indicators; and multidimensional monitoring mechanisms for continuous improvement.

#### 3.4.2. Preoperative Preparation Phase

Use structured assessments of technology acceptance and clinically relevant vulnerabilities, including frailty and fall risk [42]. Nursing supervisors coordinate local multidisciplinary teams and establish bidirectional platforms with remote supervisors, clarifying role divisions, communication protocols, and information‐sharing mechanisms. Specialized nurses formulate professional plans, circulating nurses monitor data transmission, and scrub nurses ensure robotic system compatibility.

Perform network quality assessments and audio–video verification according to technical standards. Implement standardized scenario simulation drills for emergencies (technical failures, network interruptions, and coordination barriers) to strengthen human–machine collaborative decision‐making and emergency response capacity. Postdrill debriefing may help refine cross‐regional processes and establish risk assessment records.

Complete technical status verification checklists, equipment warm‐up, personnel positioning, and preoperative team briefings to support distributed team coordination under spatial separation.

#### 3.4.3. Intraoperative Nursing Workflow

##### 3.4.3.1. Node 1: Preoperative System Verification (30 min Before Surgery)

Local and remote supervisors execute tripartite verification protocols: verify 5G network status and latency (target < 100 ms, alert 300 ms). Circulating nurses complete safety checklists confirming emergency open surgery instruments, robotic system power, and fiber optic connections. Execute WHO surgical safety checklists confirming dual‐center team readiness.

##### 3.4.3.2. Node 2: Anesthesia Induction and Positioning (Anesthesia Start to Position Fixation)

Anesthesia nurses assist in establishing vital signs monitoring and hemodynamic parameters. Circulating nurses confirm surgical positioning, ensuring anatomical landmark fixation and pressure point protection [43]. Scrub nurses complete sterile instrument preparation. Critical: patient positioning must not change after completion to avoid postdocking deviation.

##### 3.4.3.3. Node 3: Robotic System Docking (Robot Entry to Docking Completion, Target < 10 min)

Scrub nurses install robotic arm sterile covers and assist pneumoperitoneum establishment and trocar insertion. Circulating nurses confirm robotic platform positioning and imaging parameters. Remote supervisors verify console visual clarity, instrument responsiveness, and operational precision. Monitoring focus: docking represents the highest‐risk phase; strict spatial positioning and collision prevention are required.

##### 3.4.3.4. Node 4: Surgical Access Establishment (Initial Incision to Anatomical Exposure)

Scrub nurses monitor robotic arm status, preventing interarm interference and mechanical injury. Nursing supervisors establish real‐time interaction with remote supervisors. Circulating nurses monitor 5G transmission, promptly reporting latency abnormalities (> 300 ms) and image quality changes.

##### 3.4.3.5. Node 5: Critical Anatomical Operation (Organ Dissection to Vascular Processing)

Anesthesia nurses focus on hemodynamic stability, addressing blood pressure and heart rate variations. Scrub nurses precisely deliver instruments (vascular clip and sutures), minimizing response time. Nursing supervisors coordinate local teams, while remote supervisors support remote physicians.

##### 3.4.3.6. Node 6: Surgical Key Step Verification (Main Step Completion to Incision Closure)

Scrub nurses confirm sterile barrier integrity; specialized nurses verify quality indicator compliance. Circulating nurses ensure position safety (no changes during robotic connection). Remote supervisors confirm surgical completion readiness. Multidisciplinary teams jointly confirm surgical goal achievement.

##### 3.4.3.7. Node 7: System Withdrawal and Handover (Surgery End to Complete Withdrawal)

Scrub nurses support safe robotic arm withdrawal, maintaining sterility. Circulating nurses record equipment status and archive 5G connection data. Nursing supervisors implement standardized handover with remote supervisors, conduct cross‐regional quality assessments and document safety events, and establish comprehensive surgical traceability records.

##### 3.4.3.8. Node 8: Emergency Event Management (Abnormal Occurrence to Resolution)

Activate “Robotic Surgery Emergency Response Plan” with nursing supervisors commanding on‐site response. During emergency conversion, scrub nurses guide instrument repositioning and withdrawal, circulating nurses remove robotic platforms and cooperate with resuscitation, and nursing supervisors deploy emergency resources. Management principle: “patient safety first”; target an emergency response time of < 2 min.

#### 3.4.4. Postoperative Nursing Workflow

Anesthesia nurses assist anesthesiologists through technological systems in the continuous monitoring of patients’ vital signs. Professional nursing judgment, integrated with technological interfaces, enables the early identification of complications associated with remote surgery, with particular attention to risks such as mechanical injury and pneumoperitoneum‐related events. Human–machine collaborative decision‐making further supports postoperative risk assessment and intervention, with technological systems responding to nursing needs by providing real‐time monitoring data.

Nursing supervisors organize local nursing teams to perform the initial postoperative assessment, assume independent nursing responsibility for confirming stable patient vital signs, and establish postoperative collaborative mechanisms with remote healthcare teams. Cross‐regional postoperative coordination may be supported through standardized communication protocols and information‐sharing platforms, with local nursing teams serving as patient‐site representatives responsible for communicating postoperative nursing needs and supporting nursing quality monitoring.

Circulating nurses apply data transmission quality control measures to compile surgical records and technical parameters, documenting key information such as 5G transmission data, equipment status, and surgical duration to form complete surgical archives. Nursing personnel are required to continually master new standards and procedures for postoperative documentation, support consistent nursing quality under varying technological configurations and collaborative models, and apply innovative decision‐making to manage unexpected postoperative situations. This includes establishing effective mechanisms for postoperative risk identification, assessment, and response.

#### 3.4.5. Emergency Response Plans

Drawing on risk‐identification and communication‐redundancy principles discussed in the telesurgery literature, this section proposes an initial cross‐regional remote‐surgery emergency management approach [41]. Emergency plan design follows the principles of “tiered response, rapid switching, and safety priority,” with the aim of supporting patient‐safety measures and enabling rapid conversion during technical failures.

#### 3.4.6. CRSNF‐Based Nursing Quality Evaluation System Construction

Quality evaluation systems based on the three core concepts of technology‐mediated nursing, distributed nursing collaboration, and nursing adaptive capacity focus on comprehensive performance of nursing practice in remote technological environments. Technology‐mediated nursing evaluation includes 5G network transmission stability, equipment system coordination, visual adaptability, and cross‐regional information transmission accuracy, emphasizing assessment of nursing professional judgment and technological interface integration, human–machine collaboration processes, and nursing decision adaptation to technological systems. Distributed nursing collaboration evaluation encompasses multisite team coordination efficiency, emergency response capability, and cross‐cultural communication quality, reflecting implementation of role redefinition under spatial separation conditions, functioning of coordination mechanisms, and nursing team capacity for independent responsibility bearing. Nursing adaptive capacity evaluation focuses on technological learning ability, environmental adaptability, and innovative decision‐making thinking performance levels, reflecting nursing team’s continuous learning, dynamic adjustment, and problem‐solving capabilities in complex technological environments.

Descriptive observations from the two cases indicated no recorded adverse events and suggested possible differences in technological adaptation and team collaboration. These exploratory observations cannot establish statistical significance, comparative effectiveness, safety, or generalizability because they were derived from two clinically heterogeneous cases and self‐developed assessment tools. Cross‐time‐zone communication, staff adaptation, and practical verification of emergency plans remain priorities for future evaluation.

The clinical case component was limited to two clinically heterogeneous procedures from a single institution and used self‐developed indicators. No inferential statistical testing was performed. Consequently, the observed between‐case differences cannot support causal attribution, effectiveness or safety conclusions, or broad generalization. Larger multicenter prospective studies using validated instruments are needed to evaluate the framework and associated nursing outcomes.

## 4. Conclusion

This study proposed a context‐specific operational framework for remote robotic surgical nursing that translates established nursing theories into the sociotechnical workflow of cross‐regional robotic surgery. Centered on technology‐mediated nursing, distributed nursing collaboration, and nursing adaptive capacity, the preliminary CRSNF is intended to provide a structured basis for standardizing nursing processes across preoperative preparation, intraoperative management, and postoperative care, while supporting the development of quality evaluation systems. As a supporting rather than replacement framework, its effectiveness and generalizability have not been established. Multicenter prospective evaluation, expert consensus, validated instrument development, and outcome‐based assessment are required to refine the framework and determine its applicability.

## Author Contributions

Xiaodan Wu, Gang Chen, and Hua Cao formulated the research question and conceived the study. Yaling Wang and Shuang Jin contributed to the methodology. Lili Chen, Yang Yu, and Xiaofang Chen conducted the literature search. Lan Jiang oversaw the study selection process and replied to the reviewers’ comments. Yaling Wang and Lan Jiang wrote the first draft of the manuscript.

## Funding

This work was supported by the National Natural Science Foundation of China (82271238), and the National Natural Science Foundation of China Joint Fund Project (U21A20357).

## Disclosure

All authors critically reviewed and approved the final version of the manuscript.

## Ethics Statement

The systematic review component was based exclusively on previously published studies and therefore did not require ethics approval. The retrospective clinical case‐analysis component involving two remote robotic surgery cases was reviewed and approved by the Ethics Committee of Fuzhou University Affiliated Provincial Hospital (approval no. 2025–043‐01). The study was conducted in accordance with the Declaration of Helsinki.

## Conflicts of Interest

The authors declare no conflicts of interest.

## Data Availability

The data are available from the corresponding authors upon reasonable request.
